# Mortality of Philadelphia for July, August and September, 1855; Collated from the Health Office Record

**Published:** 1855-11

**Authors:** Wilson Jewell


*Mortality of Philadelphia for July, August and September*, 1855; *collated from the Health Office record.* By WILSON JEWELL, M. D.

The deaths from all causes, during the past three months, have been 3387. The period embraced within the quarter, extends through thirteen weeks, or 91 days. Making the average 37¼ deaths per day.

The most striking peculiarity, is the large decrease of deaths, compared with the corresponding quarter of last year. Amounting to 1144, or 14·45 per cent. The deaths for the third quarter of 1854, were 4531.

If we take this decrease of deaths as our guide, in forming some estimate of the health of the city during the season, knowing also that the population must have increased during the year from 2½ to 5 per cent., and that 68 of the deaths on record were from the country, it is evident, that we have enjoyed an unusual share of health.

The largest decrease seems to have been in July, viz: 677. In August it was 316 less than for August of '54, but in September it was only 151.

In the Zymotic or Endemic class of diseases, we find there was less 787, than in the same class of last year, or 25·08 per cent. In one disease alone, cholera, the difference was less 494, equal to 90·14 per cent. Dysentery, also, fell off 135 in the quarter, or 26·83 per cent.

By striking from the record the Still Born, the Accidental or External Causes, Old Age, Debility and Unknown, amounting in all to 494, we may arrive at a nearer approximation to the actual mortality for the quarter from diseases alone. If we go further, and erase from the record those from preventable diseases, we shall be able to reduce the rate still lower.

The excess of deaths in the sexes is with the males; equivalent to 7·23 per cent. In the deaths from External Causes, the excess of males is more than usually large, equal to 48·05 per cent.

Infantile mortality contributes its accustomed share in this quarter towards making up the table. We find that 1168, or 34·48 per cent. of the deaths, including the Still-Born, were under one year of age. Under five years 1898, or 56·03 per cent.

The Endemic or Zymotic class of diseases, always prevalent in the 3d quarter of the year, furnishes a large proportion of the deaths, 1175, or 34·69 per cent. Compared with the corresponding quarter of last year, there is a decrease of 25·12 per cent. Cholera Infantum in this class supplies 527. Dysentery 184; Diarrhœa 100. The deaths from Fevers amount to 152. Small Pox is evidently on the increase; as the record presents 58 deaths during the quarter. These, added to those in the first and second quarter, make 151 since the 1st of January. Not a solitary death has been recorded from measles throughout the quarter.

In the class Uncertain or General Seat, Marasmus 209, and Debility 113, make up a very large proportion of the deaths; equal to one in every one and a half of the whole number. Of Marasmus, 190 were under two years of age.

Of the Nervous affections, Convulsions in children, continue to supply a large amount of the deaths; out of 594 they furnish 212, or 35·69 per cent.

The deaths from diseases of the organs of Respiration, would be comparatively few, were it not for Consumption of the Lungs, which contributes 300, or 69·12 per cent. of the whole. During the corresponding quarter of last year, the deaths from Consumption of the Lungs were greater by 4·77 per cent. than the present one.

Old Age numbers 34 deaths. The Still Born amount to 144. This latter cause for swelling our bills of mortality weekly, appears to be on the increase instead of the decrease, as the science of medicine (or at least that branch of it which relates to Midwifery) advances.

In the table No. 3 we have the number of deaths at each of the fifteen distinct periods of life. Commencing with those under one year, we find a gradual decrease until the fifteenth year; from 1168 to 49. Beyond this period the scale is upwards until it reaches thirty, when it gradually declines to the remotest period of life.

From the Blockley Almshouse there were 210 deaths reported. Rather a frightful mortality for three months, out of a population of between two and three thousand. It will be recollected, however, that a many of those who die in the Hospital, are sent there from the different wards of the city, and oftentimes in a dying condition. Besides, the pauper population of this institution is, generally, a class with broken down health, or suffering from chronic and incurable diseases, or else bowed down by extreme old age, so that, when attacked with acute forms of disease, they seldom recover, but die within a very few days.

The unusually large number of deaths recorded as coming from the country, amounting to 67, if deducted from the aggregate for the quarter, will make the comparison between this and the same period of last year the more striking.

TABLE NO. I.

*Deaths for the third quarter of 1855 classified.*

| | | | | Male. | | | Female. | | | |
|---|---|---|---|---|---|---|---|---|---|---|
| | July | Aug. | Sept. | J. | A. | S. | J. | A. | S. | Total. |
| 1 *Endemic & Contagious diseases* Zymotic or Epidemic | 366 | 555 | 254 | 190 | 297 | 131 | 176 | 258 | 123 | 1175 |
| 2 *Uncertain or general seat,* Sporadic diseases | 142 | 206 | 135 | 80 | 111 | 64 | 62 | 95 | 71 | 483 |
| 3 Nervous system | 229 | 244 | 121 | 125 | 132 | 77 | 104 | 112 | 44 | 594 |
| 4 Organs of Respiration | 136 | 181 | 130 | 68 | 88 | 65 | 68 | 93 | 65 | 447 |
| 5 " Circulation | 20 | 19 | 12 | 9 | 11 | 6 | 11 | 8 | 6 | 51 |
| 6 Digestive organs | 65 | 93 | 46 | 30 | 49 | 26 | 35 | 44 | 20 | 204 |
| 7 Urinary " | 1 | 4 | 1 | | 2 | | 1 | 2 | 1 | 6 |
| 8 Organs of Generation | 4 | 9 | 5 | | | | 4 | 9 | 5 | 18 |
| 9 " Locomotion | 5 | 4 | | 4 | 2 | | 1 | 2 | | 9 |
| 10 Integumentary system | 3 | | 3 | 1 | | 2 | 2 | | 1 | 6 |
| 11 Old age | 5 | 16 | 13 | 2 | 7 | 5 | 3 | 9 | 8 | 34 |
| 12 External causes | 59 | 61 | 34 | 44 | 42 | 28 | 15 | 19 | 6 | 154 |
| Still Born | 54 | 46 | 44 | 30 | 27 | 26 | 24 | 19 | 18 | 144 |
| Unknown | 24 | 17 | 21 | 13 | 11 | 11 | 11 | 6 | 10 | 62 |
| | 1113 | 1455 | 819 | 596 | 779 | 441 | 517 | 676 | 378 | 3387 |

### TABLE NO. 2.

1. *Endemic and Contagious Diseases—Zymotic or Epidemic.*

| | Male. | Female. | Under 1 yr. | 1 to 2. | 2 to 5. | 5 to 10. | 10 to 15. | 15 to 20. | 20 to 30. | 30 to 40. | 40 to 50. | 50 to 60. | 60 to 70. | 70 to 80. | 80 to 90. | 90 to 100. | 100 to 110. | Total. |
|---|---|---|---|---|---|---|---|---|---|---|---|---|---|---|---|---|---|---|
| Cholera | 8 | 19 | | | | 1 | 1 | | 4 | 6 | 5 | 1 | 5 | 3 | 1 | | | 27 |
| " infantum | 288 | 239 | 363 | 147 | 17 | | | | | | | | | | | | | 527 |
| " morbus | 7 | 15 | 4 | | 2 | | | | 4 | 6 | 1 | 3 | | 2 | | | | 22 |
| Croup | 18 | 20 | 5 | 7 | 19 | 7 | | | | | | | | | | | | 38 |
| Diarrhœa | 50 | 50 | 48 | 17 | 8 | | 1 | 1 | 5 | 6 | 2 | 7 | 2 | 3 | | | | 100 |
| Dysentery | 99 | 85 | 36 | 27 | 25 | 6 | 4 | 8 | 20 | 16 | 11 | 10 | 13 | 4 | 4 | | | 184 |
| Erysipelas | 7 | 3 | 4 | | 2 | 1 | | 1 | 1 | | 1 | | | | | | | 10 |
| Fever, | 4 | 2 | | 2 | | | 1 | | | 1 | 1 | 1 | | | | | | 6 |
| " Bilious | 4 | 7 | | | | | | 2 | | 3 | 1 | 3 | | 2 | | | | 11 |
| " Congestive | 3 | | | | 1 | | | 1 | | 1 | | | | | | | | 3 |
| " Intermittent | 1 | 2 | | | 1 | | | | | | | | | | | 1 | 1 | 3 |
| " Malignant | 1 | | | | | | | | 1 | | | | | | | | | 1 |
| " Nervous | | 1 | | | | | | | | 1 | | | | | | | | 1 |
| " Pernicious | 2 | | | | | | | | 1 | | | | | 1 | | | | 2 |
| " Remittent | 6 | 9 | 5 | 1 | 1 | | 1 | 1 | 2 | | | | | 1 | 2 | 1 | | 15 |
| " Scarlet | 14 | 12 | 3 | 3 | 14 | 6 | | | | | | | | | | | | 26 |
| " Typhoid | 27 | 32 | 2 | | 5 | 1 | 2 | 10 | 12 | 11 | 9 | 2 | 4 | 1 | | | | 59 |
| " Typhus | 13 | 10 | | | | 1 | 1 | 3 | 10 | 5 | | | 1 | 1 | 1 | | | 23 |
| " Yellow | 2 | | | | | | | 1 | | | 1 | | | | | | | 2 |
| Hooping Cough | 27 | 17 | 19 | 17 | 7 | 1 | | | | | | | | | | | | 44 |
| Influenza | | 1 | 1 | | | | | | | | | | | | | | | 1 |
| Small Pox | 33 | 25 | 9 | 7 | 19 | 10 | 1 | 3 | 4 | 2 | 1 | 2 | | | | | | 58 |
| Syphilis | | 2 | 2 | | | | | | | | | | | | | | | 2 |
| Varicella | 1 | | 1 | | | | | | | | | | | | | | | 1 |
| Varioloid | 3 | 5 | 1 | 1 | 1 | 2 | 1 | | 1 | 1 | | | | | | | | 8 |
| | 618 | 556 | 503 | 229 | 122 | 36 | 13 | 31 | 65 | 59 | 33 | 29 | 25 | 18 | 8 | 2 | 1 | 1174 |

### 2. *Uncertain or General Seat—Sporadic Diseases.*

| | Male. | Female. | Under 1 yr. | 1 to 2. | 2 to 5. | 5 to 10. | 10 to 15. | 15 to 20. | 20 to 30. | 30 to 40. | 40 to 50. | 50 to 60. | 60 to 70. | 70 to 80. | 80 to 90. | 90 to 100. | 100 to 110. | Total. |
|---|---|---|---|---|---|---|---|---|---|---|---|---|---|---|---|---|---|---|
| Abscess | 4 | 1 | 3 | | 1 | 1 | | | | | | | | | | | | 5 |
| " Thigh | | 1 | | | | | | | | 1 | | | | | | | | 1 |
| Angina Faucium | | 1 | | | 1 | | | | | | | | | | | | | 1 |
| Cancer | 5 | 6 | | | | 1 | | | 1 | 1 | 1 | 3 | | 4 | | | | 11 |
| " Breast | | 2 | | | | | | | | | | 2 | | | | | | 2 |
| Carbuncle, Malignant | 1 | | | | | | | | | | | 1 | | | | | | 1 |
| Cyanosis | 6 | 4 | 10 | | | | | | | | | | | | | | | 10 |
| Cachexia | | 1 | | 1 | | | | | | | | | | | | | | 1 |
| Debility | 56 | 57 | 65 | 4 | | 3 | | 1 | 2 | 4 | 8 | 5 | 10 | 5 | 4 | 2 | | 113 |
| Dropsy | 18 | 21 | 1 | | 4 | 1 | | | 2 | 2 | 4 | 5 | 9 | 6 | 5 | | | 39 |
| Gangrene | 4 | 3 | | | | | | | 1 | | 1 | 1 | 2 | | 2 | | | 7 |
| Hemorrhage | 3 | 1 | 1 | 1 | | | | | | 1 | | 1 | | | | | | 4 |
| Fungus Hematodes | 1 | | | | | | | | 1 | | | | | | | | | 1 |
| Inflammation | 1 | | | 1 | | | | | | | | | | | | | | 1 |
| " Throat | 2 | 3 | 3 | | 1 | | | | | | | | | 1 | | | | 5 |
| " Tonsils | | 1 | | 1 | | | | | | | | | | | | | | 1 |
| Inanition | 24 | 13 | 31 | | 1 | | | | | | 3 | | 2 | | | | | 37 |
| Malformation | 6 | 6 | 12 | | | | | | | | | | | | | | | 12 |
| Marasmus | 113 | 96 | 141 | 49 | 11 | 2 | 1 | | 1 | 1 | | 1 | 1 | 1 | | | | 209 |
| Mortification | | 2 | 1 | 1 | | | | | | | | | | | | | | 2 |
| Osteo Sarcoma | | 1 | | | | | | | | 1 | | | | | | | | 1 |
| Scrofula | 8 | 7 | 3 | 2 | 2 | 1 | 2 | | 3 | 1 | | 1 | | | | | | 15 |
| Ulceration | 1 | | | | | | | | | 1 | | | | | | | | 1 |
| " Throat | 2 | 1 | 1 | | | 2 | | | | | | | | | | | | 3 |
| | 255 | 228 | 272 | 60 | 21 | 11 | 3 | 1 | 11 | 13 | 17 | 20 | 24 | 17 | 11 | 2 | | 483 |

### 3. *Nervous Diseases.*

| | Male. | Female. | Under 1 yr. | 1 to 2. | 2 to 5. | 5 to 10. | 10 to 15. | 15 to 20. | 20 to 30. | 30 to 40. | 40 to 50. | 50 to 60. | 60 to 70. | 70 to 80. | 80 to 90. | 90 to 100. | 100 to 110. | Total. |
|---|---|---|---|---|---|---|---|---|---|---|---|---|---|---|---|---|---|---|
| Abscess of Brain | 1 | | | | 1 | | | | | | | | | | | | | 1 |
| Apoplexy | 10 | 14 | 1 | | | 1 | | | 1 | 6 | 1 | 2 | 6 | 5 | | 1 | | 24 |
| Congestion of Brain | 34 | 23 | 20 | 7 | 8 | 6 | 3 | 2 | 2 | 3 | 3 | 2 | | 1 | | | | 57 |
| Compression " | 1 | | 1 | | | | | | | | | | | | | | | 1 |
| Convulsions | 112 | 100 | 138 | 47 | 17 | 7 | | | 3 | | | | | | | | | 212 |
| " Puerperal | | 4 | | | | | | | 3 | 1 | | | | | | | | 4 |
| Coup de Soliel | 11 | 2 | | | | | | 1 | 4 | 5 | | 1 | 1 | 1 | | | | 13 |
| Disease of Brain | 17 | 12 | 6 | 6 | 5 | 1 | 1 | 1 | 1 | 2 | 1 | 2 | 2 | 1 | | | | 29 |
| Dropsy " | 42 | 33 | 35 | 25 | 12 | 2 | 1 | | | | | | | | | | | 75 |
| Disease of Nerves | 1 | 1 | 1 | | | | | | 1 | | | | | | | | | 2 |
| Effusion of Brain | 16 | 13 | 12 | 14 | 2 | 1 | | | | | | | | | | | | 29 |
| Epilepsy | 3 | 5 | 2 | 1 | | | 1 | | 1 | 2 | 1 | | | | | | | 8 |
| Inflammation of Brain | 54 | 33 | 29 | 27 | 13 | 7 | 2 | 3 | 3 | | 2 | 1 | | | | | | 87 |
| Mania | 2 | 1 | | | | | | | 1 | | | 1 | 1 | | | | | 3 |
| " a Potu | 6 | | | | | | | | | 5 | 1 | | | | | | | 6 |
| Nervous Erethism | | 1 | | | | | | | 1 | | | | | | | | | 1 |
| Palsy | 10 | 15 | | | 1 | | | | 4 | 1 | 4 | 7 | 2 | 4 | 1 | 1 | | 25 |
| Softening of Brain | 7 | | 1 | 3 | | | | | | | 1 | 1 | | 1 | | | | 7 |
| Tetanus | 4 | 2 | | 1 | | | 1 | 2 | 2 | | | | | | | | | 6 |
| Trismus | 3 | 1 | 2 | | 1 | | | | | | | | 1 | | | | | 4 |
| | 334 | 260 | 248 | 131 | 60 | 25 | 9 | 9 | 27 | 25 | 14 | 17 | 13 | 13 | 1 | 2 | | 594 |

### 4. *Organs of Respiration.*

| | Male. | Female. | Under 1 yr. | 1 to 2. | 2 to 5. | 5 to 10. | 10 to 15. | 15 to 20. | 20 to 30. | 30 to 40. | 40 to 50. | 50 to 60. | 60 to 70. | 70 to 80. | 80 to 90. | 90 to 100. | 100 to 110. | Total. |
|---|---|---|---|---|---|---|---|---|---|---|---|---|---|---|---|---|---|---|
| Abscess of Lungs | | 2 | | | | | | | 1 | 1 | | | | | | | | 2 |
| Apoplexy " | | 1 | | | | | | | | | | | 1 | | | | | 1 |
| Asthma | 2 | 2 | | | | | | | | 1 | 1 | | 1 | 1 | | | | 4 |
| Congestion of Lungs | 3 | 9 | 6 | | 2 | 1 | | | 1 | | | 1 | | 1 | | | | 12 |
| Consumption " | 150 | 159 | 11 | 3 | 7 | 1 | 6 | 28 | 106 | 64 | 50 | 15 | 11 | 7 | | | | 309 |
| " Laryngeal | | 1 | | | | | | | | | 1 | | | | | | | 1 |
| Coryza | | 1 | 1 | | | | | | | | | | | | | | | 1 |
| Disease of Lungs | 5 | | 1 | | | | | 1 | 2 | | 1 | | | | | | | 5 |
| " Chest | 5 | 3 | | | 3 | | | | 1 | | | 1 | 3 | | | | | 8 |
| Dropsy of " | 5 | 4 | 1 | | | 2 | 2 | | | 2 | | | 2 | | | | | 9 |
| Hectic Fever | | 1 | | | | | | | 1 | | | | | | | | | 1 |
| Hemorrhage of Lungs | 3 | 3 | | | | | | | 1 | 3 | 1 | 1 | | | | | | 6 |
| Inflamm'n of Bronchiæ | 14 | 10 | 10 | 4 | 2 | 3 | | | | 1 | 1 | | | 2 | 1 | | | 24 |
| " Larynx | 3 | | | | 1 | 1 | | | | | | 1 | | | | | | 3 |
| " Lungs | 30 | 30 | 16 | 14 | 8 | 3 | 1 | 4 | 2 | 6 | 1 | 1 | 1 | 1 | 2 | | | 60 |
| " Pleura | 1 | 1 | | | | | | | | 1 | | | 1 | | | | | 2 |
| | 221 | 227 | 46 | 21 | 23 | 11 | 9 | 33 | 115 | 79 | 56 | 20 | 20 | 12 | 3 | | | 448 |
| Still Born | 83 | 61 | 144 | | | | | | | | | | | | | | | 144 |
| Unknown | 35 | 27 | 24 | 5 | 1 | 1 | 1 | 2 | 5 | 8 | 6 | 4 | 4 | 1 | | | | 62 |

### 5. *Organs of Circulation.*

| | Male. | Female. | Under 1 yr. | 1 to 2. | 2 to 5. | 5 to 10. | 10 to 15. | 15 to 20. | 20 to 30. | 30 to 40. | 40 to 50. | 50 to 60. | 60 to 70. | 70 to 80. | 80 to 90. | 90 to 100. | 100 to 110. | Total. |
|---|---|---|---|---|---|---|---|---|---|---|---|---|---|---|---|---|---|---|
| Anæmia | 3 | 2 | 4 | | | | | | | 1 | | | | | | | | 5 |
| Angina Pectoris | | 1 | | | | | | | | | | | | 1 | | | | 1 |
| Disease of Heart | 20 | 15 | 1 | 1 | | 1 | 2 | 2 | 6 | 3 | 4 | 5 | 8 | 2 | | | | 35 |
| Dropsy " | 2 | 3 | | | | | | | 1 | | 1 | 1 | | 2 | | | | 5 |
| Enlargem't " | 1 | 2 | | | 1 | | | | 1 | | | 1 | | | | | | 3 |
| Inflammat'n " | | 2 | | | 1 | | | | | 1 | | | | | | | | 2 |
| | 26 | 25 | 5 | 1 | 2 | 1 | 2 | 2 | 8 | 5 | 5 | 7 | 8 | 5 | | | | 51 |

### 6. *Digestive Organs.*

| | Male. | Female. | Under 1 yr. | 1 to 2. | 2 to 5. | 5 to 10. | 10 to 15. | 15 to 20. | 20 to 30. | 30 to 40. | 40 to 50. | 50 to 60. | 60 to 70. | 70 to 80. | 80 to 90. | 90 to 100. | 100 to 110. | Total. |
|---|---|---|---|---|---|---|---|---|---|---|---|---|---|---|---|---|---|---|
| Cancer of Stomach & Bowels | | 1 | | | | | | | | | | | | | 1 | | | 1 |
| " Stomach | 2 | | | | | | | | | 1 | | | | 1 | | | | 2 |
| Cancrum Oris | 1 | 1 | | | 2 | | | | | | | | | | | | | 2 |
| Colic | | 3 | | 1 | | | | | 1 | | | 1 | | | | | | 3 |
| Disease of Bowels | 1 | | | | | | | | | | | | 1 | | | | | 1 |
| " Liver | 4 | 4 | | | | | | | | 1 | 2 | 3 | 1 | | 1 | | | 8 |
| " Stomach | | 1 | | | 1 | | | | | | | | | | | | | 1 |
| Dropsy Abdominal | 4 | 5 | | 1 | | 1 | | | | | 3 | 1 | 2 | | 1 | | | 9 |
| Dyspepsia | 2 | 1 | 2 | | | | | | | | 1 | | | | | | | 3 |
| Enlargement of Liver | 1 | | | | | | 1 | | | | | | | | | | | 1 |
| Gangrene of Intestines | 1 | | | | | | | | | 1 | | | | | | | | 1 |
| Hemorrhage of Bowels | | 1 | | | | | | | | 1 | | | | | | | | 1 |
| " Stomach | 2 | 2 | | | | | | | 1 | 1 | 1 | | | 1 | | | | 4 |
| " Umbilicus | | 1 | 1 | | | | | | | | | | | | | | | 1 |
| Hernia | | 2 | 1 | | | | | | | | 1 | | | | | | | 2 |
| " Strangulated | | 1 | | | | | | | | 1 | | | | | | | | 1 |
| Ileus | 2 | 1 | 1 | | | | | | | | | | 1 | 1 | | | | 3 |
| Induration of Stomach | 1 | | | | | | | | | | 1 | | | | | | | 1 |
| Inflammation of Bowels | 21 | 25 | 14 | 11 | 2 | 1 | 1 | 1 | 2 | 5 | | 3 | 4 | 1 | | 1 | | 46 |
| " Liver | 7 | 4 | 1 | | 1 | | | 1 | 2 | 3 | 1 | 1 | 1 | | | | | 11 |
| " Peritoneum | 2 | 5 | | | 1 | 1 | 1 | 1 | 2 | | | 1 | | | | | | 7 |
| " Stomach | 10 | 11 | 6 | 2 | | | | | | 5 | 2 | 2 | 2 | 2 | | | | 21 |
| " Stom. & Bowels | 6 | 5 | 2 | 1 | 1 | | | | | 1 | 2 | 1 | 2 | | 1 | | | 11 |
| Intussusception | 2 | | 1 | | 1 | | | | | | | | | | | | | 2 |
| Jaundice | 6 | 5 | 4 | | | | | 1 | | | 1 | 3 | 1 | 1 | | | | 11 |
| Obstruction of Bowels | 1 | 2 | | | | | 1 | | | | 1 | | | 1 | | | | 3 |
| " Liver | 1 | | | | | | | | | | 1 | | | | | | | 1 |
| Perforation of Intestines | 1 | | | | | | | | | | | | 1 | | | | | 1 |
| Stricture of Esophagus | 1 | | | | | | | | | | | | | 1 | | | | 1 |
| Sore Mouth | 5 | 3 | 8 | | | | | | | | | | | | | | | 8 |
| Tabes Mesenterica | 7 | 7 | 10 | 2 | 2 | | | | | | | | | | | | | 14 |
| Teething | 7 | 4 | 3 | 7 | 1 | | | | | | | | | | | | | 11 |
| Ulceration of Intestines | 1 | 4 | 2 | 1 | | | | | | 2 | | | | | | | | 5 |
| " Liver | 1 | | | | | | | | | | | 1 | | | | | | 1 |
| " Stomach | 1 | 1 | | 1 | | | | | | 1 | | | | | | | | 2 |
| Worms | 1 | 2 | 1 | 1 | | | | | 1 | | | | | | | | | 3 |
| | 105 | 99 | 57 | 28 | 12 | 3 | 4 | 4 | 9 | 23 | 17 | 17 | 16 | 9 | 4 | 1 | | 204 |

### 7. *Urinary Organs.*

| | Male. | Female. | Under 1 yr. | 1 to 2. | 2 to 5. | 5 to 10. | 10 to 15. | 15 to 20. | 20 to 30. | 30 to 40. | 40 to 50. | 50 to 60. | 60 to 70. | 70 to 80. | 80 to 90. | 90 to 100. | 100 to 110. | Total. |
|---|---|---|---|---|---|---|---|---|---|---|---|---|---|---|---|---|---|---|
| Albuminuria | 1 | 3 | | | | | | 1 | 2 | | | | | 1 | | | | 4 |
| Diabetes | 1 | | | | | | | | | 1 | | | | | | | | 1 |
| Inflamed Bladder | | 1 | | | | | | | | | 1 | | | | | | | 1 |
| | 2 | 4 | | | | | | 1 | 2 | 1 | 1 | | | 1 | | | | 6 |

8. *Organs of Generation.*

| | Male. | Female. | Under 1 yr. | 1 to 2. | 2 to 5. | 5 to 10. | 10 to 15. | 15 to 20. | 20 to 30. | 30 to 40. | 40 to 50. | 50 to 60. | 60 to 70. | 70 to 80. | 80 to 90. | 90 to 100. | 100 to 110. | Total. |
|---|---|---|---|---|---|---|---|---|---|---|---|---|---|---|---|---|---|---|
| Cancer of Uterus | | 5 | | | | | | | | 2 | 2 | 1 | | | | | | 5 |
| Hemorrhage of Uterus | | 6 | | | | | | | 2 | 2 | 2 | | | | | | | 6 |
| Inflammation " | | 3 | | | | | | | 2 | | 1 | | | | | | | 3 |
| Ovarian Tumor | | 1 | | | | | | | | | 1 | | | | | | | 1 |
| Rupture of Uterus | | 2 | | | | | | | | 2 | | | | | | | | 2 |
| Schirrhus " | | 1 | | | | | | | | | | | 1 | | | | | 1 |
| | | 18 | | | | | | | 4 | 6 | 6 | 1 | 1 | | | | | 18 |

9. *Organs of Locomotion.*

| | Male. | Female. | Under 1 yr. | 1 to 2. | 2 to 5. | 5 to 10. | 10 to 15. | 15 to 20. | 20 to 30. | 30 to 40. | 40 to 50. | 50 to 60. | 60 to 70. | 70 to 80. | 80 to 90. | 90 to 100. | 100 to 110. | Total. |
|---|---|---|---|---|---|---|---|---|---|---|---|---|---|---|---|---|---|---|
| Caries of Spine | 1 | | | | | | | | 1 | | | | | | | | | 1 |
| Disease of " | 1 | | | | 1 | | | | | | | | | | | | | 1 |
| " Hip | 1 | 2 | | 1 | | 1 | | | | 1 | | | | | | | | 3 |
| Inflammation of Spine | 1 | | | 1 | | | | | | | | | | | | | | 1 |
| Rheumatism | 2 | | | | | | | | | 2 | | | | | | | | 2 |
| Spina Bifida | | 1 | 1 | | | | | | | | | | | | | | | 1 |
| | 6 | 3 | 1 | 2 | 1 | 1 | | | 1 | 3 | | | | | | | | 9 |

10. *Integumentary System.*

| | Male. | Female. | Under 1 yr. | 1 to 2. | 2 to 5. | 5 to 10. | 10 to 15. | 15 to 20. | 20 to 30. | 30 to 40. | 40 to 50. | 50 to 60. | 60 to 70. | 70 to 80. | 80 to 90. | 90 to 100. | 100 to 110. | Total. |
|---|---|---|---|---|---|---|---|---|---|---|---|---|---|---|---|---|---|---|
| Herpes | | 1 | | 1 | | | | | | | | | | | | | | 1 |
| Hives | | 1 | 1 | | | | | | | | | | | | | | | 1 |
| Purpura Hemorrhagica | 1 | 2 | 2 | 1 | | | | | | | | | | | | | | 3 |
| Rash | | 1 | | 1 | | | | | | | | | | | | | | 1 |
| | 1 | 5 | 3 | 3 | | | | | | | | | | | | | | |

11. *Old Age.*

| | Male. | Female. | Under 1 yr. | 1 to 2. | 2 to 5. | 5 to 10. | 10 to 15. | 15 to 20. | 20 to 30. | 30 to 40. | 40 to 50. | 50 to 60. | 60 to 70. | 70 to 80. | 80 to 90. | 90 to 100. | 100 to 110. | Total. |
|---|---|---|---|---|---|---|---|---|---|---|---|---|---|---|---|---|---|---|
| Old Age | 14 | 20 | | | | | | | | | | | | 10 | 16 | 7 | 1 | 34 |

12. *External Causes.*

| | Male. | Female. | Under 1 yr. | 1 to 2. | 2 to 5. | 5 to 10. | 10 to 15. | 15 to 20. | 20 to 30. | 30 to 40. | 40 to 50. | 50 to 60. | 60 to 70. | 70 to 80. | 80 to 90. | 90 to 100. | 100 to 110. | Total. |
|---|---|---|---|---|---|---|---|---|---|---|---|---|---|---|---|---|---|---|
| Asphyxia | 5 | 2 | 6 | | | | | | | 1 | | | | | | | | 7 |
| Burns | 3 | | 1 | 2 | | | | | | | | | | | | | | 3 |
| Casualties | 40 | 9 | 1 | 1 | | 1 | 2 | 3 | 13 | 12 | 12 | 3 | | 1 | | | | 49 |
| Drowned | 32 | 11 | | 1 | 2 | 7 | 6 | 3 | 10 | 10 | 3 | | | 1 | | | | 43 |
| Effects of Heat | 11 | 4 | | | | | | | 1 | 5 | 3 | 2 | 2 | 1 | 1 | | | 15 |
| Exhaustion | 1 | 2 | | | | | | | | 1 | 1 | | | 1 | | | | 3 |
| Exposure | 1 | | | | | | | | | 1 | | | | | | | | 1 |
| Fracture | 2 | 3 | | | | | | | | 2 | | 1 | 1 | | 1 | | | 5 |
| Hydrophobia | 1 | | | | | | | | | | | 1 | | | | | | 1 |
| Intemperance | 8 | 6 | | | | | | | 1 | 5 | 5 | 3 | | | | | | 14 |
| Neglect | 1 | | 1 | | | | | | | | | | | | | | | 1 |
| Poisoning | 1 | 1 | | 1 | | | | | | | | 1 | | | | | | 2 |
| Suffocation | 4 | | | | 1 | | | | 1 | 1 | 1 | | | | | | | 4 |
| Suicide | 4 | 2 | | | | | | | 1 | | 1 | 1 | 2 | 1 | | | | 6 |
| | 1 4 | 40 | 9 | 5 | 3 | 8 | 8 | 6 | 27 | 38 | 26 | 12 | 5 | 5 | 2 | | | 154 |

### TABLE NO. 3.

*Deaths for the Third Quarter of 1855, at fifteen distinct periods of life.*

| | |
|---|---|
| Under 1 year, | 1168 |
| 1 to 2 | 485 |
| 2 to 5 | 245 |
| 5 to 10 | 97 |
| 10 to 15 | 49 |
| 15 to 20 | 89 |
| 20 to 30 | 274 |
| 30 to 40 | 260 |
| 40 to 50 | 181 |
| 50 to 60 | 127 |
| 60 to 70 | 116 |
| 70 to 80 | 91 |
| 80 to 90 | 45 |
| 90 to 100 | 14 |
| 100 to 110 | 2 |
| | 3243 |
| Still Born | 144 |
| Total, | 3387 |

Embraced within the above table were 210 from the Blockley Alms House; Blacks, 174, and from the country 67, as follows:

| | July. | Aug. | Sept. | Total. |
|---|---|---|---|---|
| Almshouse, | 65 | 92 | 53 | 210 |
| Blacks, | 56 | 81 | 37 | 174 |
| Country, | 16 | 24 | 27 | 67 |
| | 137 | 197 | 117 | 451 |

